# KREMEN1 residue W94 is essential for receptor binding and infection by a major group of enteroviruses

**DOI:** 10.1128/mbio.01123-26

**Published:** 2026-07-31

**Authors:** Xiaohong Li, Pan Liu, Hongzheng Li, Youwei Zhu, Ruiyi Zhang, Xingchen Xie, Rui Yu, Feilong Zhou, Jingjing Yan, Min Wang, Jiayi Shu, Chenli Qiu, Shuo Zhang, Zichen Yang, Zhijun Liu, Xiaoyan Zhang, Jianqing Xu, Chao Zhang, Shuye Zhang

**Affiliations:** 1 Clinical Center for Biotherapy, Zhongshan Hospital, Fudan University 12478, Shanghai, China; 2 Shanghai Institute of Infectious Disease and Biosecurity, Fudan University 12478, Shanghai, China; 3 State Key Laboratory of Virology and Hubei Province Key Laboratory of Allergy and Immunology, and Department of Immunology, Wuhan University TaiKang Medical School (School of Basic Medical Sciences) 36841, Wuhan, China; 4 Institutes of Biomedical Sciences, Fudan University 12478, Shanghai, China; 5 Shanghai Public Health Clinical Center, Fudan University 12478, Shanghai, China; 6 Engineering Research Center of Cell & Therapeutic Antibody, School of Pharmacy, Shanghai Jiao Tong University 12474, Shanghai, China; 7 Department of Microbiology, Weifang Medical University 372527, Weifang, China; The Ohio State University, Columbus, Ohio, USA

**Keywords:** hand, foot, and mouth disease, enterovirus, KREMEN1 receptor, infection

## Abstract

KREMEN1 (KRM1) serves as a cellular receptor for a major group of enteroviruses causing hand, foot, and mouth disease (HFMD), including CVA2–CVA6, CVA8, CVA10, and CVA12. The viral VP2 residue K140 has recently been identified as completely conserved among these viruses and critical for interaction with KRM1 and viral infection. However, the molecular determinants on the receptor side that govern broad enterovirus recognition remain incompletely understood. Here, we identify KRM1 tryptophan 94 (W94) as an essential residue for KRM1-mediated enterovirus infection. Alanine scanning of 29 KRM1 residues at the CVA10–KRM1 interface revealed that only W94A nearly abolished CVA10 infection. Structural analysis revealed that KRM1 W94 engages in a π-cation interaction with the viral VP2 residue K140, which is critical for receptor binding. Unlike wild-type mice, which succumbed to CVA10 infection, CRISPR-engineered mouse Krm1 W94A mutant mice were fully resistant. Moreover, the W94A mutation conferred universal resistance to all KRM1-dependent enteroviruses (CVA2–CVA6, CVA8, CVA10, and CVA12), both in cell culture and *in vivo*, indicating that the W94 residue is essential for infection by these viruses. Collectively, these findings establish KRM1 W94 as a critical receptor determinant that pairs with the universally conserved VP2 K140 to enable enterovirus infection. This work provides a structural and mechanistic basis for the broad recognition of KRM1-dependent enteroviruses and offers insights that may guide the future development of broad-spectrum antiviral strategies.

**IMPORTANCE:**

Hand, foot, and mouth disease affects millions of children worldwide each year; however, no effective antiviral drugs are available. Many causative viruses, including coxsackievirus A10 and A6, rely on the cellular receptor KREMEN1 to enter human cells. However, which parts of the receptor are critical for viral binding and infection have remained unclear. Here, we identified tryptophan 94 (W94) of KREMEN1 as critical for viral infection. Introducing the W94A mutation abolished viral binding and infection. Structural analysis revealed that W94 interacts directly with a completely conserved residue K140 on the viral capsid protein VP2. Notably, mice carrying this mutation were completely resistant to all KREMEN1-dependent viruses. These findings provide a molecular basis for virus-receptor recognition and highlight a promising target for broad-spectrum therapies against hand, foot, and mouth disease.

## INTRODUCTION

Species A enteroviruses (EV-As) comprise multiple serotypes, including coxsackieviruses CVA2–CVA8, CVA10, CVA12, CVA14, CVA16 and enterovirus A71 (EV-A71) (1, 2). These viruses cause a spectrum of diseases, such as hand, foot, and mouth disease (HFMD), herpangina, and severe neurological complications, seriously threatening the health of infants and young children (3–5). In the past, enterovirus A71 (EV-A71) was a major pathogen, but its prevalence has now declined substantially (6). Currently, coxsackievirus A6 (CVA6) and coxsackievirus A10 (CVA10) have emerged as the leading causes of HFMD outbreaks worldwide (4, 7, 8). The licensed inactivated EV-A71 vaccines do not provide cross-protection against other circulating enteroviruses (9). In addition, no specific antiviral drugs are available for HFMD. Therefore, a deep understanding of viral entry into host cells is critical for developing broadly effective preventive and therapeutic strategies.

Enterovirus entry is mediated by specific interactions between the viral capsid and cellular receptors. A major group of EV-As, including CVA2, CVA3, CVA4, CVA5, CVA6, CVA8, CVA10, and CVA12, utilizes KREMEN1 (KRM1) as a functional receptor (10, 11). Structural analyses of the CVA10-KRM1 and CVA6-KRM1 complexes have shown that KRM1 binds to the canyon region of the viral capsid, contacting residues from VP1, VP2, and VP3 (12–14). In particular, the VP2 EF-loop plays a critical role in receptor engagement (11, 15). We recently demonstrated that VP2 residue K140 within this loop is completely conserved across all KRM1-dependent enteroviruses and serves as a universal anchor for KRM1 binding (11). However, the essential determinants on the receptor side have remained largely unknown. Specifically, the KRM1 residues that enable broad recognition of multiple enteroviruses remain to be identified.

In this study, we systematically investigated the functional contributions of 29 KRM1 residues at the CVA10-KRM1 interface through alanine-scanning mutagenesis. We identified a single residue, tryptophan 94 (W94), that is essential for KRM1-mediated viral infection. We further characterized the effect of the W94A mutation on receptor binding, viral attachment, and infectivity in cell culture. We also generated CRISPR-engineered mice carrying the Krm1 W94A mutation to evaluate its role *in vivo*. Our results reveal that KRM1 W94 pairs with the conserved viral VP2 residue K140 through a π-cation interaction and is indispensable for infection by all KRM1-dependent enteroviruses.

## RESULTS

### KRM1 W94 residue is identified as essential for CVA10 infection

**Fig 1:**
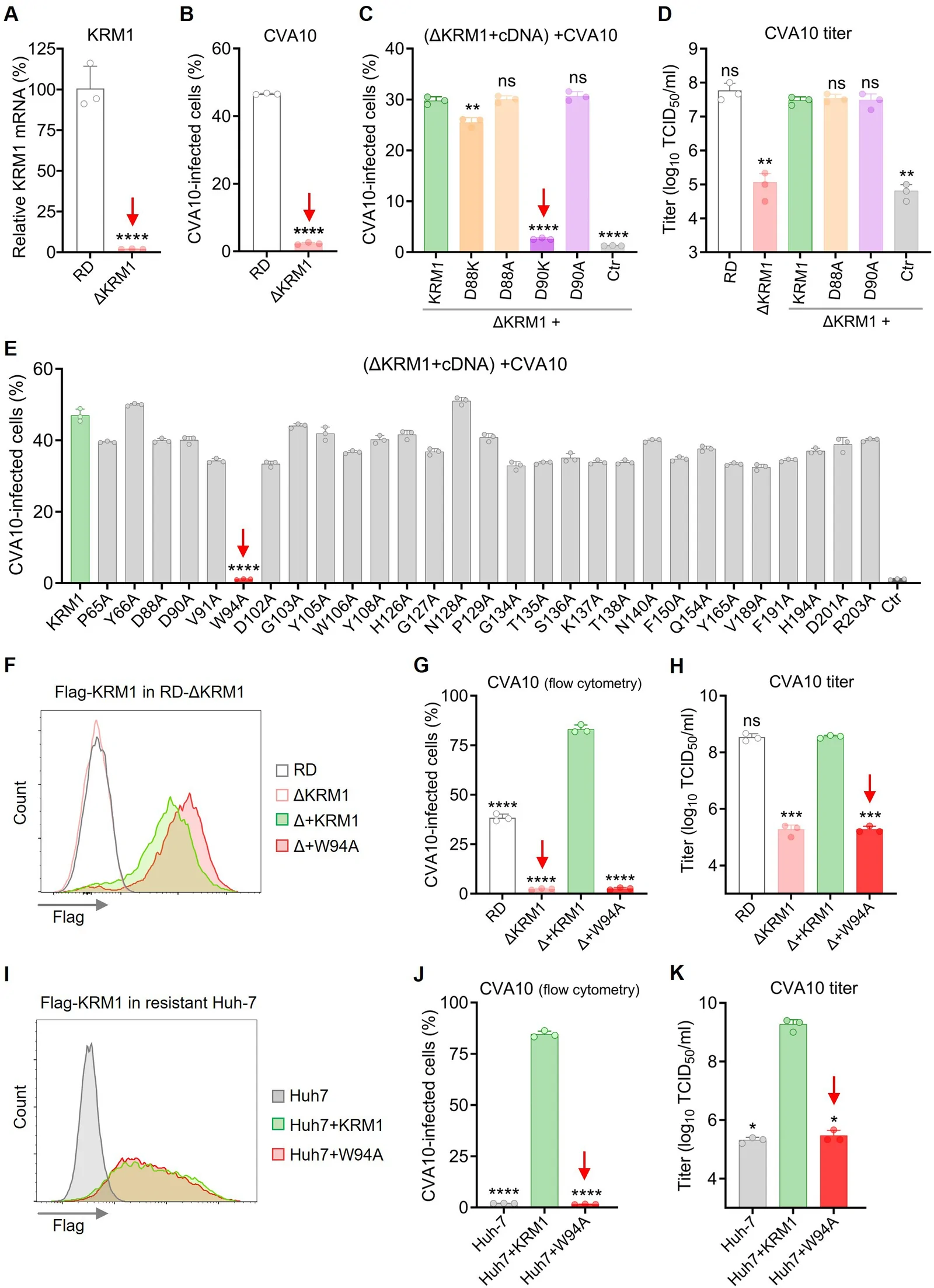
The KRM1 W94 residue is essential for CVA10 infection. (**A**) RT-qPCR analysis of KRM1 mRNA levels in RD and ΔKRM1 cells. Data were presented as mean ± SD from three biological replicates, normalized to β-actin mRNA. (**B**) RD and ΔKRM1 cells were infected with CVA10 at a multiplicity of infection (MOI) of 1 for 6 h, and CVA10 infection was measured by flow cytometry using J2 antibody. Data are shown as mean ± SD from three biological replicates. (**C**) ΔKRM1 cells were transfected with cDNA of SCARB2 (control, Ctr), KRM1-WT, or the indicated KRM1 mutants and then infected with CVA10 at an MOI of 1 for 6 h. The cells were stained with J2 antibody, and the percentage of CVA10-infected cells was analyzed by flow cytometry. Data are mean ± SD from three biological replicates. Downward arrow indicates the KRM1 D90K substitution that impaired CVA10 infection. (**D**) Cells transfected as in panel **C** were infected with CVA10 at an MOI of 0.01 for 24 h, and progeny virus titers were quantified by TCID_50_ assay. Data are mean ± SD from three biological replicates. (**E**) Screening of KRM1 residues critical for CVA10 infection. ΔKRM1 cells expressing each mutant were infected with CVA10 (MOI = 1) for 6 h, and CVA10 infection was measured by flow cytometry. Downward arrows indicate W94A, the only mutation that impaired infection. Data are mean ± SD from three biological replicates. (**F**) Flow cytometric detection of total Flag-tagged KRM1 expression in RD, ΔKRM1, Δ+KRM1, and Δ+W94 A cells. RD cells stained with an isotype control antibody served as the control. (**G**) RD, ΔKRM1, Δ+KRM1, and Δ+W94 A cells were infected with CVA10 at an MOI of 1 for 6 h, CVA10-positive cells were quantified by flow cytometry. Data are mean ± SD from three biological replicates. (**H**) The same cells as in panel **F** were infected with CVA10 at an MOI of 0.01 for 24 h, and viral titers were determined by TCID_50_ assay. Data are mean ± SD from three biological replicates. (**I**) Flow cytometric analysis of total Flag-tagged KRM1 expression in Huh-7, Huh7+KRM1, and Huh7+W94 A cells. Huh-7 cells stained with an isotype control antibody served as the control. (**J**) Huh-7, Huh7+KRM1, and Huh7+W94 A cells were infected with CVA10 at an MOI of 1 for 6 h, and the percentage of CVA10-positive cells was analyzed by flow cytometry. Data are mean ± SD from three biological replicates. (**K**) The same cells as in panel **J** were infected with CVA10 at an MOI of 0.01 for 24 h, and progeny virus titers were measured by TCID_50_ assay. Data are mean ± SD from three biological replicates. Statistical significance was determined by a two-tailed unpaired *t*-test. *, *P* < 0.05; **, *P* < 0.01; ***, *P* < 0.001; ****, *P* < 0.0001; ns, not significant (*P* ≥ 0.05).

To identify the KRM1 residues critical for CVA10 infection, we first generated KRM1-knockout RD cells (ΔKRM1) using CRISPR/Cas9 with two sgRNAs targeting exon 2 of the KRM1 gene (Fig. S1). Sequencing of the edited locus revealed that one allele carried two single-base deletions (one in each sgRNA target site), while the other allele contained a larger 68-bp deletion spanning both sgRNA regions (Fig. S1). Consequently, KRM1 mRNA levels were markedly reduced in ΔKRM1 cells compared to wild-type RD cells, as determined by reverse-transcription quantitative PCR (RT-qPCR) (Fig. 1A). As expected, CVA10 infection was severely impaired in ΔKRM1 cells, as measured by flow cytometric analysis of viral dsRNA to monitor viral replication (Fig. 1B), confirming the successful disruption of KRM1.

Previous studies showed that the conserved VP2 residue K140 of CVA10 forms hydrogen bonds and salt bridges with KRM1 residues D88 and D90 (11–13). Charge-reversal mutations (D88K and D90K) differentially affected viral infection: D90K nearly abolished infection, whereas D88K had only a minor effect (11). However, such charge-reversal substitutions are drastic and may overestimate the functional contribution of these residues. To assess their intrinsic roles without charge inversion, we generated neutral alanine substitutions (D88A and D90A). ΔKRM1 cells expressing wild-type KRM1, D88K, D90K, D88A, or D90A were infected with CVA10. Flow cytometric analysis of CVA10-positive cells revealed that the D90K mutation markedly reduced infection, while the D88K mutation had only a minimal effect (Fig. 1C), consistent with our previous report (11). Notably, neither the D88A nor the D90A mutation impaired viral infection (Fig. 1C). To confirm that the alanine substitutions indeed had no impact on viral replication, we measured progeny virus titers. Consistent with the flow cytometry results, both D88A and D90A produced viral titers comparable to wild-type KRM1 (Fig. 1D). Thus, the charge-reversal mutation D90K may overstate the role of this residue, suggesting that there may be other KRM1 residue(s) at the interface that are critical for viral entry.

Based on the cryo-EM structure of the CVA10-KRM1 complex, 29 KRM1 residues are predicted to contact the virus (12, 13). To systematically identify which of these residues are functionally important, we substituted each with alanine and tested the ability of the mutants to support CVA10 infection in ΔKRM1 cells. As shown in Fig. 1E, alanine substitutions at most positions had little or no effect on infection. Strikingly, only the W94A mutation almost completely abolished viral infection (Fig. 1E). To validate this finding, we reconstituted ΔKRM1 cells with either wild-type KRM1 or the W94A mutant (designated Δ+KRM1 and Δ+W94A, respectively). Flow cytometry confirmed the comparable expression of Flag-tagged KRM1-WT and KRM1-W94A in these cells (Fig. 1F). When infected with CVA10, Δ+KRM1 cells regained susceptibility, whereas Δ+W94A cells remained resistant, similar to ΔKRM1 cells (Fig. 1G). Consistently, progeny virus titers were readily detected in Δ+KRM1 cells, whereas those in Δ+W94A cells remained as low as in ΔKRM1 cells (Fig. 1H). We extended these observations to Huh-7 cells, which are naturally resistant to CVA10. We generated Huh7+KRM1 and Huh7+W94A cell lines by lentiviral transduction. Flow cytometry confirmed comparable Flag-tagged expression of KRM1-WT and KRM1-W94A (Fig. 1I). Parental Huh-7 cells were resistant to CVA10 infection, showing only a low percentage of CVA10-positive cells and low progeny virus titers (Fig. 1J–K). Expression of KRM1-WT in Huh7+KRM1 cells markedly increased the percentage of CVA10-positive cells (Fig. 1J) and progeny virus titers (Fig. 1K), rendering them susceptible to infection. In contrast, Huh7+W94A cells remained largely resistant, with infection levels comparable to those of parental Huh-7 cells (Fig. 1J and K). Collectively, these results identify KRM1 W94 as an essential residue for KRM1-mediated CVA10 infection.

### Alanine mutations of KRM1 residues near the conserved VP2 residue K140 do not alter receptor biochemical properties or structure

**Fig 2:**
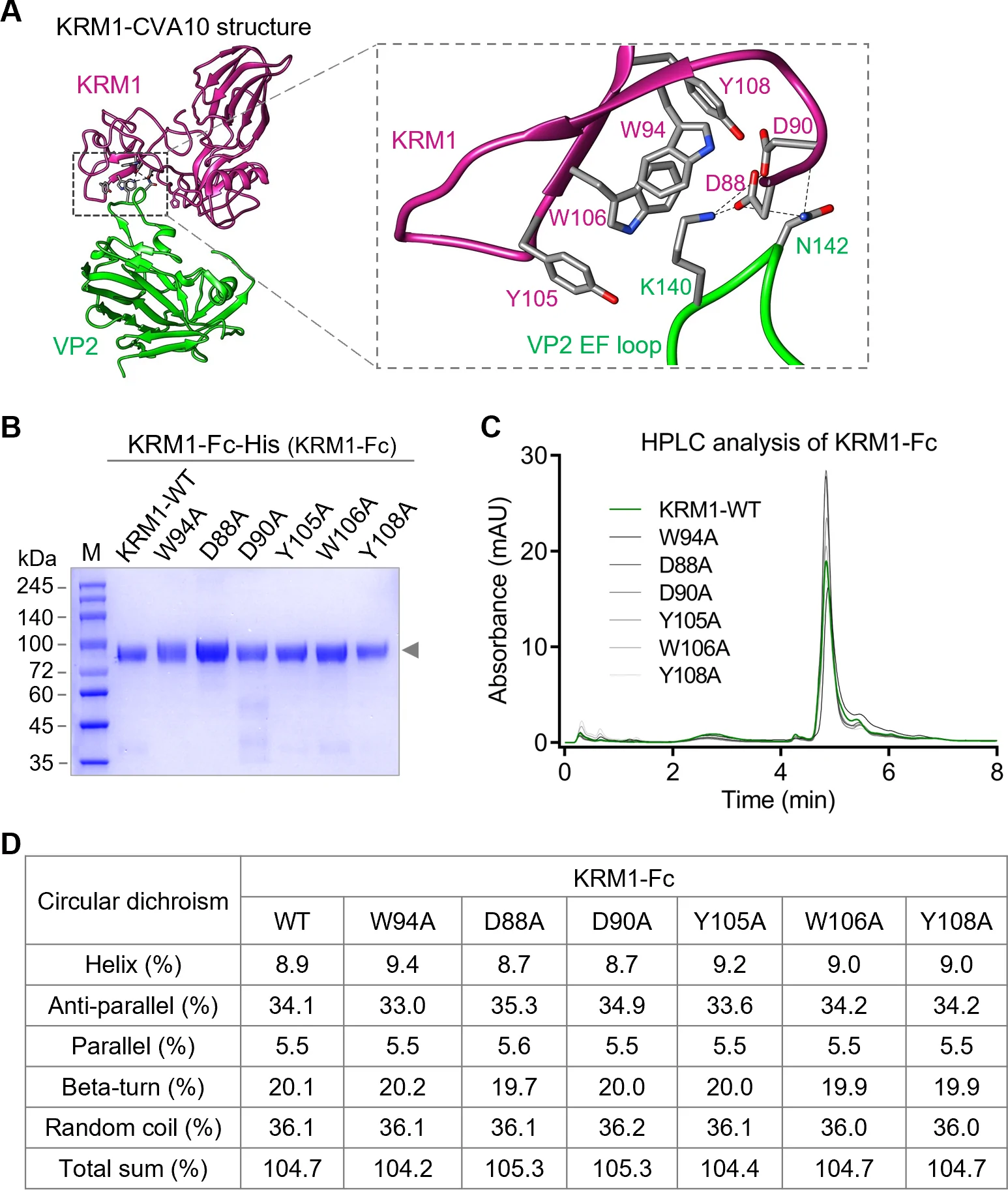
Alanine mutations of KRM1 residues near the conserved VP2 residue K140 do not alter receptor biochemical properties or structure. (**A**) Structural model showing the interface between CVA10 and KRM1 (PDB: 7BZU). VP2 residues K140 and N142 are spatially adjacent to KRM1 residues D88, D90, W94, Y105, W106, and Y108. KRM1, violet red. VP2-EF loop, green. Nitrogen atom, blue; oxygen, red; carbon, gray. Dashed lines indicate hydrogen bonds. (**B**) SDS-PAGE analysis of purified KRM1-WT-Fc-His and KRM1-Fc mutant proteins. Each lane was loaded with 3 µg of protein. M, protein marker. (**C**) HPLC chromatograms of purified KRM1 WT-Fc-His and KRM1-Fc mutant proteins. (**D**) CD spectra of KRM1 WT-Fc-His and the indicated mutant proteins. Summary of secondary structure proportions of KRM1 WT and mutants.

Previous studies identified VP2 residues N142 and K140 as critical for CVA10-KRM1 interaction, with K140 being completely conserved and serving as a universal anchor for KRM1 binding (11, 15). Given the critical role of VP2 K140, we selected a set of KRM1 residues surrounding it, including D88, D90, W94, Y105, W106, and Y108 (Fig. 2A), to test whether they contribute to receptor-virus binding. We expressed and purified wild-type KRM1 and the corresponding alanine mutants (D88A, D90A, W94A, Y105A, W106A, and Y108A) as Fc-fusion proteins. SDS-PAGE analysis confirmed that all purified proteins exhibited the expected molecular weights and similar migration patterns, with high purity (Fig. 2B).

To assess protein hydrophobicity and oligomeric state, we performed high-performance liquid chromatography (HPLC) on a C3 reverse-phase column. All tested proteins eluted as single symmetric peaks with comparable retention times (Fig. 2C), indicating that none of the mutations altered the hydrophobic properties or oligomeric state of the KRM1 protein. We further analyzed the secondary structure by circular dichroism (CD) spectroscopy. The CD spectra and the calculated secondary structure proportions were similar for wild-type and all mutant proteins (Fig. 2D). Collectively, these biophysical analyses demonstrate that the tested alanine mutations do not affect the overall structure or biochemical properties of KRM1. Therefore, the impaired infection caused by the W94A mutation (Fig. 1) is unlikely to result from global misfolding but rather reflects the disruption of a specific receptor-virus contact.

### KRM1 W94 is a key functional residue required for the CVA10-KRM1 interaction

**Fig 3:**
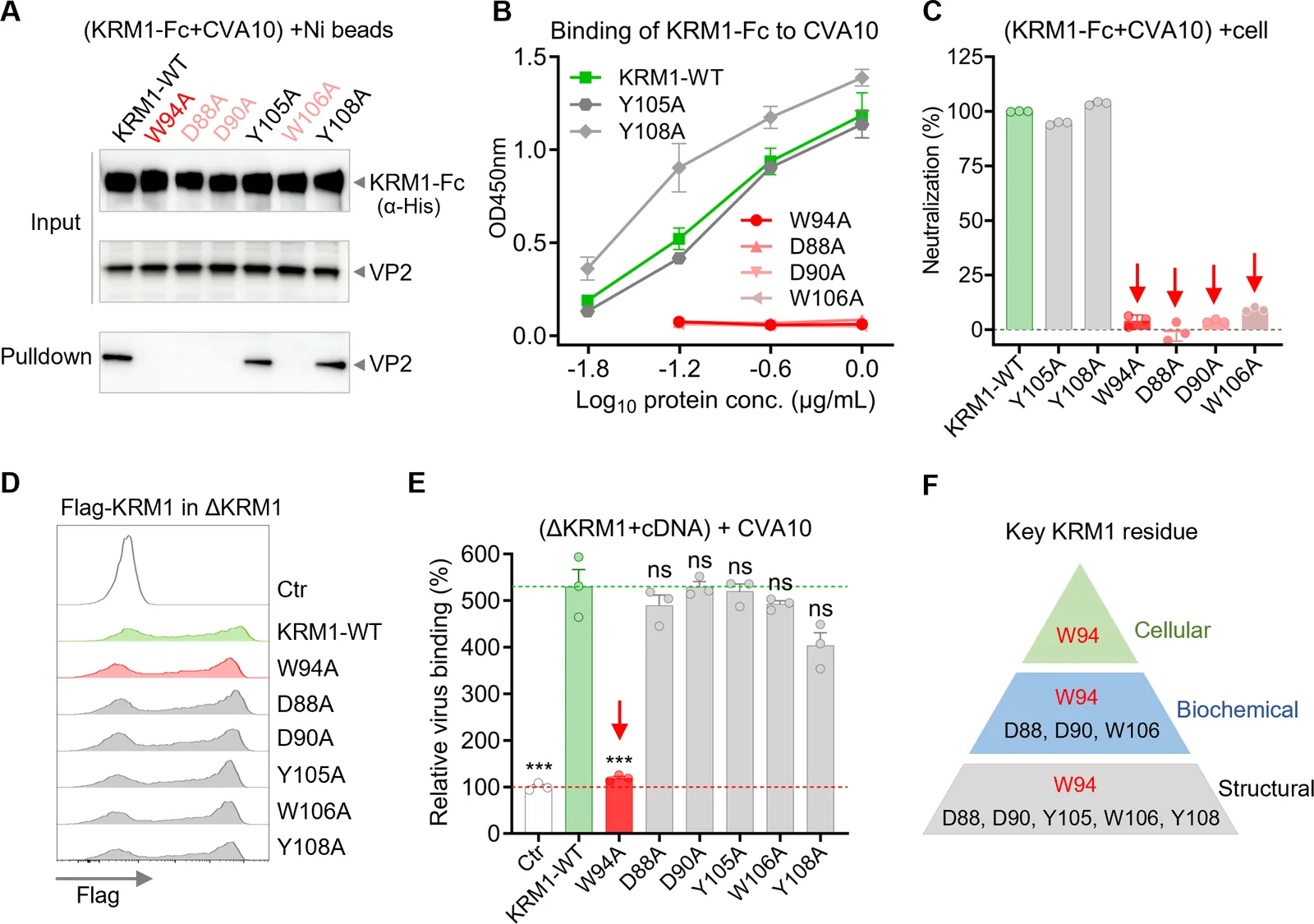
KRM1 W94 is a key functional residue required for the CVA10-KRM1 interaction. (**A**) Western blot analysis of CVA10 incubated with KRM1-WT-Fc-His, or the indicated KRM1 mutant proteins. CVA10 was detected using an anti-VP2 antibody, while KRM1-WT-Fc-His and mutant proteins were detected with an anti-His antibody. Dark red and light red indicate the KRM1 mutant protein is unable to bind CVA10. This experiment was performed twice, with similar results. (**B**) ELISA analysis of CVA10 binding to KRM1-WT-Fc-His and mutant proteins. Purified CVA10 virions were coated on ELISA plates and incubated with serial dilutions of each protein, followed by detection with HRP-conjugated goat anti-human IgG. Data are presented as mean ± SD. (**C**) Receptor protein neutralization assay. CVA10 (MOI = 1) was preincubated with KRM1-WT-Fc-His, or mutant proteins and the mixtures were added to RD cells. Cells were stained with anti-dsRNA antibody, and CVA10-positive cells were analyzed by flow cytometry at 6 hpi. The neutralizing activity of KRM1-WT-Fc-His protein against CVA10 was normalized to 100%. Data are mean ± SD of three biological replicates. Note that red downward arrows denote KRM1 proteins unable to effectively neutralize CVA10 infection. (**D**) ΔKRM1 cells transfected with KRM1-WT or the indicated mutant cDNAs were analyzed by flow cytometry for Flag-KRM1 protein expression. (**E**) Analysis of the binding of CVA10 to transfected cells by RT-qPCR. ΔKRM1 cells were transfected with SCARB2 (Ctr), KRM1-WT, or the indicated mutant cDNA for 24 h, followed by incubation with CVA10 (MOI = 5) on ice for 1 h. The bound CVA10 was detected by RT-qPCR, normalized to β-actin mRNA levels. The viral RNA bound to ΔKRM1 cells transfected with SCARB2 (Ctr) was defined as 100%. Data are presented as mean ± SD from three biological replicates. Statistical analysis was performed using a two-tailed unpaired t-test comparing KRM1-complemented ΔKRM1 cells with other groups. ***, *P* < 0.001; ns, no significant difference (*P* ≥ 0.05). Red downward arrows denote that the KRM1 W94A mutation impaired CVA10 binding to cells. (**F**) Summary of KRM1 residues located at the CVA10-KRM1 interface and their functional contributions to viral binding and infection.

To evaluate the contribution of KRM1 residues near the conserved VP2 residue K140 to virus-receptor binding, we tested the same panel of alanine mutants (D88A, D90A, W94A, Y105A, W106A, and Y108A) for their ability to bind CVA10. Pull-down assays showed that wild-type KRM1 strongly bound CVA10, as detected by the VP2 band in western blot (Fig. 3A). In contrast, the W94A, D88A, D90A, and W106A mutations disrupted CVA10 binding, whereas Y105A and Y108A had no detectable effect (Fig. 3A). These results were confirmed by receptor-binding ELISA using purified CVA10 virions as the coated antigen (Fig. 3B). The W94A, D88A, D90A, and W106A mutations abolished binding, whereas Y105A and Y108A retained binding. Consistent with the binding data, neutralization assays revealed that wild-type KRM1-Fc, KRM1-Y105A-Fc, and KRM1-Y108A-Fc efficiently neutralized CVA10 infection, while the W94A, D88A, D90A, and W106A mutants failed to neutralize (Fig. 3C).

To evaluate the functional relevance of these residues in a cellular context, we transfected ΔKRM1 cells with cDNAs encoding wild-type KRM1 or each alanine mutant. Flow cytometry confirmed comparable expression of all Flag-tagged proteins (Fig. 3D). We then performed a cell-based virus attachment assay by incubating the transfected cells with CVA10 at 4°C and quantifying bound viral RNA by RT-qPCR (Fig. 3E). Expression of wild-type KRM1 markedly enhanced viral binding to the cell surface. Similarly, the D88A, D90A, Y105A, W106A, and Y108A mutants all increased binding to levels comparable to wild-type KRM1. In striking contrast, the W94A mutation failed to enhance binding, remaining at the control level (Fig. 3E). These results highlight that although multiple KRM1 residues can engage CVA10 *in vitro*, only W94 is essential for virus attachment at the cellular level. A summary of the functional contributions of these KRM1 residues is presented in Fig. 3F. Thus, KRM1 W94 represents the key functional residue for KRM1-mediated infection.

### Krm1 W94A mutant mice are resistant to CVA10 infection

**Fig 4:**
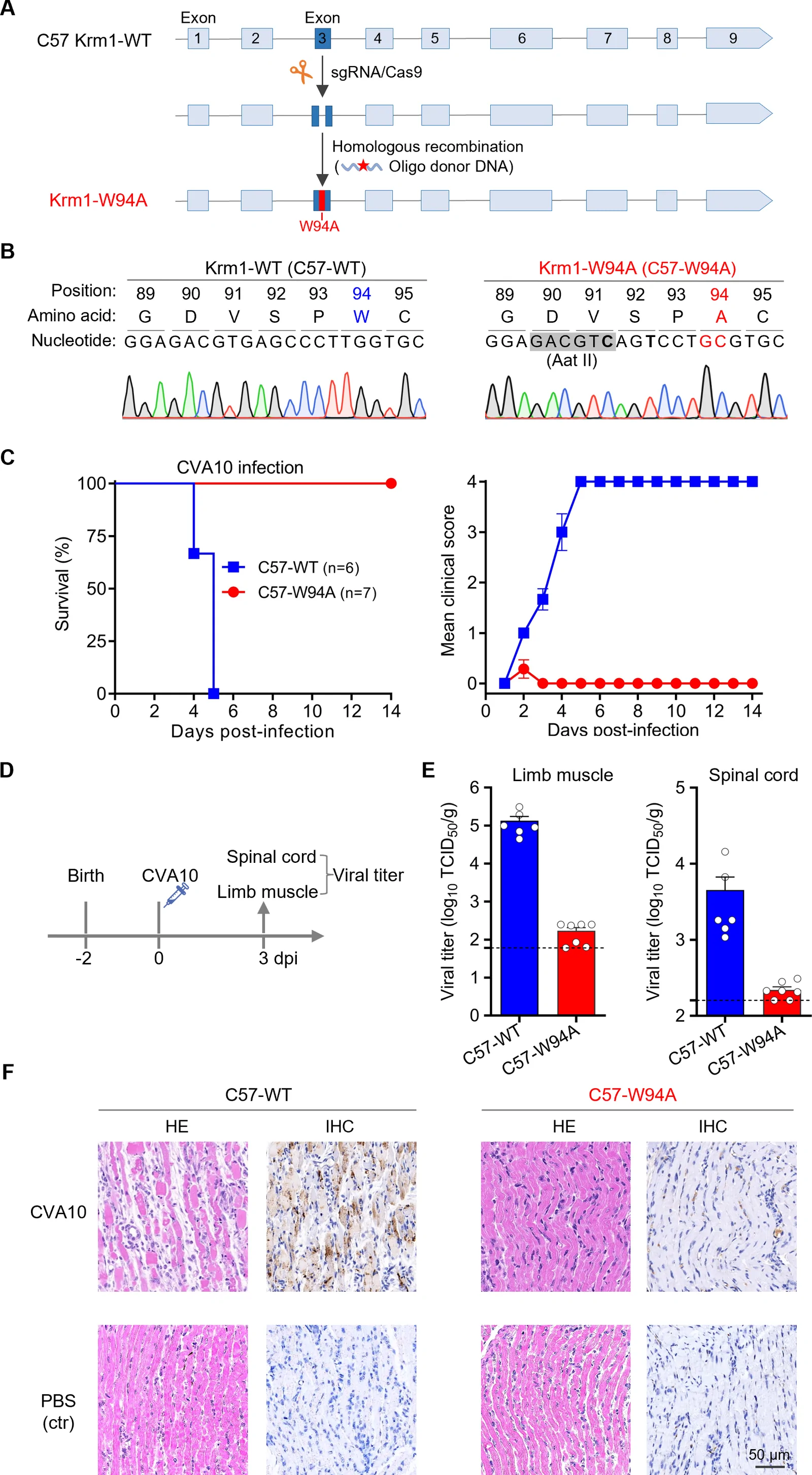
Krm1 W94A mutant mice are resistant to CVA10 infection. (**A**) Construction strategy for Krm1 W94A mutant mice. The KRM1 W94A mutation was introduced into C57BL/6 mice via CRISPR/Cas9-mediated homology-directed repair (HDR), as described in the Methods section. (**B**) Sequence chromatograms of the edited genomic regions from C57-WT and C57-W94A, confirming the successful introduction of the W94A mutation. The restriction site *Aat II* created by the synonymous mutation is shaded in gray. (**C**) Survival and clinical signs of C57-WT (blue line) and C57-W94A (red line) following intraperitoneal infection with CVA10. Groups of 2-day-old mice were inoculated with 1 × 10^5^ TCID_50_ of CVA10, and then survival and clinical signs were observed daily. Clinical scores were graded as follows: 0, healthy; 1, reduced mobility; 2, limb weakness; 3, limb paralysis; and 4, death. Group sizes (N) are indicated; data are means ± SEM. (**D**) Schematic of CVA10 intraperitoneal (i.p.) infection in mice for viral titer determination. (**E**) Two-day-old C57-WT and C57-W94A mice were challenged with CVA10, and viral titers in limb muscles and spinal cord were measured, as depicted in (**D**). The dashed line indicates the limit of detection (LOD), which was 60 TCID_50_/g tissue for the hind limb muscle and 160 TCID_50_/g tissue for the spinal cord. Data are means ± SD. A two-tailed unpaired *t*-test was used to compare the differences. **, *P* < 0.01; ***, *P* < 0.001. (**F**) Two-day-old C57-WT and C57-W94A neonatal mice were challenged with CVA10 or PBS, and limb muscle tissues were collected at 3 dpi for hematoxylin and eosin (H&E) staining and immunohistochemical analysis using an anti-dsRNA antibody. Scale bar, 50 μm.

To evaluate the *in vivo* function of KRM1 W94 for CVA10 infection, we first analyzed the sequence conservation between human KRM1 (hKRM1) and mouse KRM1 (mKrm1). Sequence alignment revealed that hKRM1 and mKrm1 proteins are highly conserved overall, especially within the Kringle and WSC domains that participate in CVA10 binding (Fig. S2A). Moreover, complementation assays in ΔKRM1 cells showed that mKrm1, like its human counterpart, effectively restored CVA10 infection, and this activity was abolished by the W94A mutation (Fig. S2B and C). These results validated the use of the mouse model for studying W94 function *in vivo*. We therefore generated C57BL/6J mice carrying the Krm1 W94A mutation via CRISPR/Cas9-mediated homologous recombination (Fig. 4A). Sanger sequencing of the targeted genomic region confirmed the successful substitution of tryptophan 94 with alanine (Fig. 4B). The Krm1 W94A mice developed normally and were fertile, compared to wild-type controls.

Two-day-old wild-type (C57-WT) and Krm1 W94A (C57-W94A) mice were intraperitoneally infected with CVA10. All C57-WT mice died within 4–5 days post-infection, whereas C57-W94A mice remained completely healthy and survived throughout the observation period (Fig. 4C). At 3 days post-infection (dpi), viral titers in the limb muscle and spinal cord were measured by TCID_50_ assay (Fig. 4D). C57-WT mice had high viral loads in limb muscle (geometric mean titer: 1.1 × 10⁵ TCID_50_/g tissue). In contrast, viral titers in C57-W94A mice were near the limit of detection (142 TCID_50_/g tissue), representing a 767-fold reduction (Fig. 4E). In the spinal cord, C57-WT mice had a geometric mean titer of 2.8 × 10³ TCID_50_/g tissue, while C57-W94A mice showed 214 TCID_50_/g tissue, a 13-fold reduction (Fig. 4E). Consistently, hematoxylin and eosin (H&E) staining of limb muscle from CVA10-infected C57-WT mice showed extensive myofiber necrosis, whereas muscle sections from infected C57-W94A mice appeared normal, similar to PBS-treated controls (Fig. 4F). Immunohistochemical (IHC) staining for dsRNA with J2 antibody on limb muscle of CVA10-infected mice revealed strong viral replication signals in C57-WT tissue, but no detectable signal in C57-W94A mice (Fig. 4F). These results demonstrate that the KRM1 W94 residue is critical for CVA10 pathogenesis *in vivo*.

### The π-cation interaction between KRM1 W94 and CVA10 VP2 residue K140 is critical for receptor binding and infection

**Fig 5:**
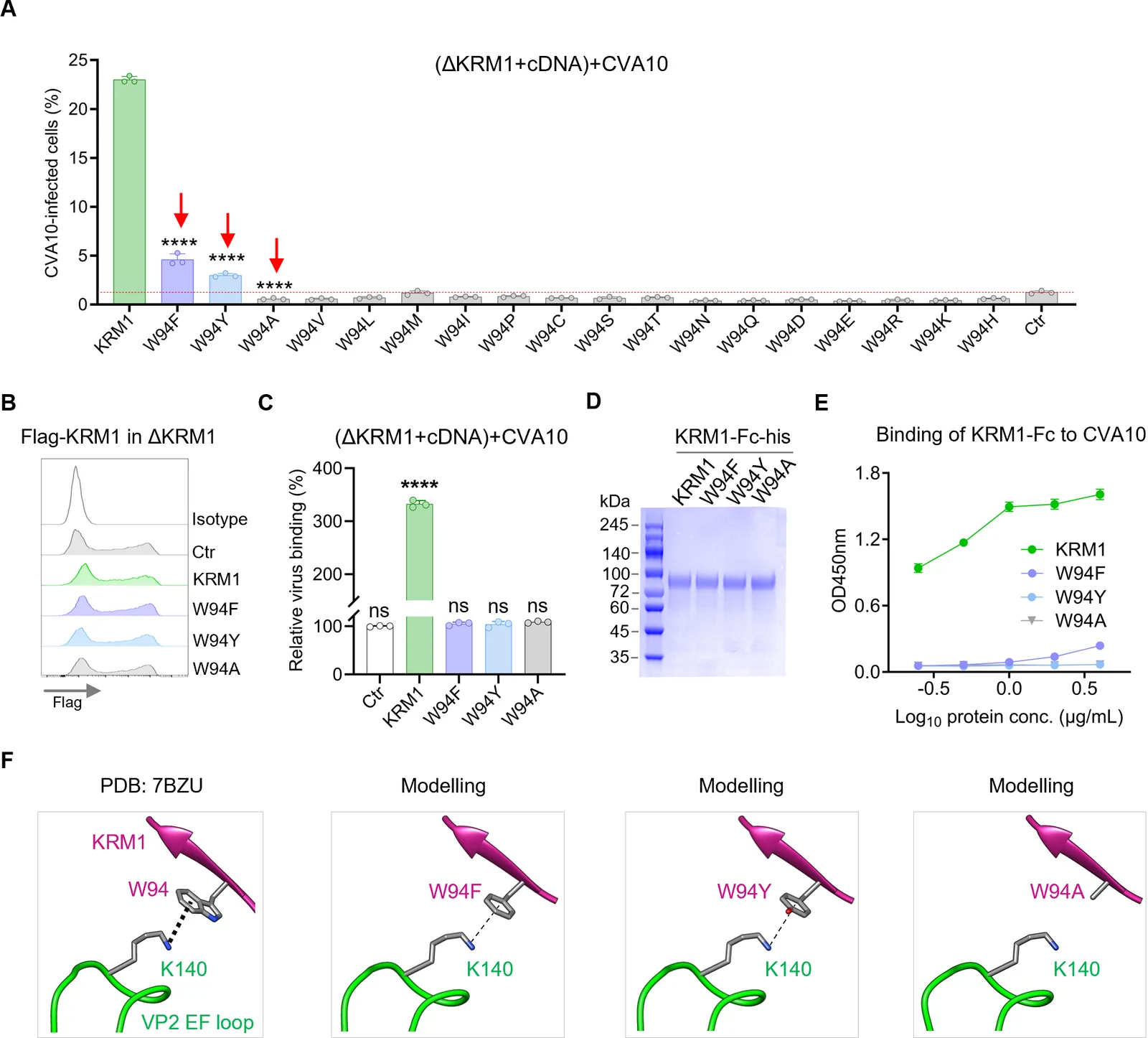
The π-cation interaction between KRM1 W94 and CVA10 VP2 residue K140 is critical for receptor binding and infection. (**A**) ΔKRM1 cells expressing ctr (SCARB2), KRM1, or the indicated KRM1 mutant cDNAs were infected with CVA10 (MOI = 0.3). At 6 hpi, the cells were stained with J2 antibody and anti-Flag antibody and were analyzed by flow cytometry for the percentage of CVA10-infected cells. Data are mean ± SD of three biological replicates. (**B**) ΔKRM1 cells transfected with KRM1-WT or the indicated mutant cDNAs were analyzed by flow cytometry for Flag-KRM1 protein expression. (**C**) RT-qPCR was performed to assess the binding of CVA10 to ΔKRM1 cells reconstituted with KRM1-WT or the indicated KRM1 mutants. The CVA10 bound to ΔKRM1 cells transfected with SCARB2 (Ctr) was defined as 100%. Data are mean ± SD of three biological replicates. Statistical significance was determined by the two-tailed *t*-test to assess differences. ****, *P* < 0.0001; ns, no significant difference. (**D**) Purified Fc fusion proteins, including KRM1-WT, KRM1 W94F, KRM1 W94Y, and KRM1 W94A (2 μg each), were separated by SDS-PAGE and stained with Coomassie Brilliant Blue. (**E**) ELISA analysis of CVA10 binding to KRM1-WT-Fc and mutant proteins. Purified CVA10 virions were coated on ELISA plates and incubated with serial dilutions of each protein, followed by detection with HRP-conjugated goat anti-human IgG. Data are presented as mean ± SD. (**F**) The impact of the mutation of KRM1 W94 on the π-cation interaction was analyzed using SWISS-MODEL. Nitrogen atom, blue; oxygen, red; carbon, gray. The thick black dashed line represents the strongest π-cation interaction between W94 and K140.

To investigate the mechanism by which KRM1 W94 supports CVA10 infection, we performed saturation mutagenesis at residue W94. ΔKRM1 cells expressing wild-type KRM1 or the W94F, W94Y, W94A, or other substitutions were infected with CVA10. Wild-type KRM1 supported infection in 23% of the cells. The W94F and W94Y mutants allowed low-level infection (4.6% and 3.0%, respectively), whereas all other mutations, including W94A, failed to restore the infection (Fig. 5A). Flow cytometry confirmed that wild-type KRM1, W94F, W94Y, and W94A were expressed at comparable levels in ΔKRM1 cells (Fig. 5B). Cell-based binding assays showed that expression of wild-type KRM1 markedly enhanced CVA10 attachment to the cell surface, whereas W94F, W94Y, and W94A all failed to increase binding above the control level (Fig. 5C).

To examine the direct virus-receptor binding, we purified the ectodomains of KRM1-WT, KRM1-W94F, KRM1-W94Y, and KRM1-W94A as Fc-fusion proteins. All proteins showed similar migration on SDS-PAGE (Fig. 5D). Receptor-binding ELISA using purified CVA10 virions as the coated antigen revealed that wild-type KRM1 bound CVA10 in a concentration-dependent manner (Fig. 5E). The W94F mutant showed only weak binding at the highest concentration tested (4 μg/mL), whereas W94Y and W94A exhibited no detectable binding (Fig. 5E). These results indicate that the loss of binding capacity among KRM1 W94 mutants correlates with the severity of the infection defect (Fig. 5A).

We next examined the structural basis of this interaction. Analysis of the published CVA10-KRM1 cryo-EM structure (PDB: 7BZU) revealed a π-cation interaction between the side chain of KRM1 W94 and the side chain of viral VP2 residue K140 (Fig. 5F) (12). Homology modeling indicated that the W94F and W94Y substitutions could retain a weakened π-cation interaction with K140, whereas W94A could not form such an interaction (Fig. 5F). Collectively, these data demonstrate that the π-cation interaction between KRM1 W94 and CVA10 VP2 residue K140 is critical for receptor binding and infection.

### The KRM1 W94 residue is crucial for both cellular binding and infection of all KRM1-dependent enteroviruses

**Fig 6:**
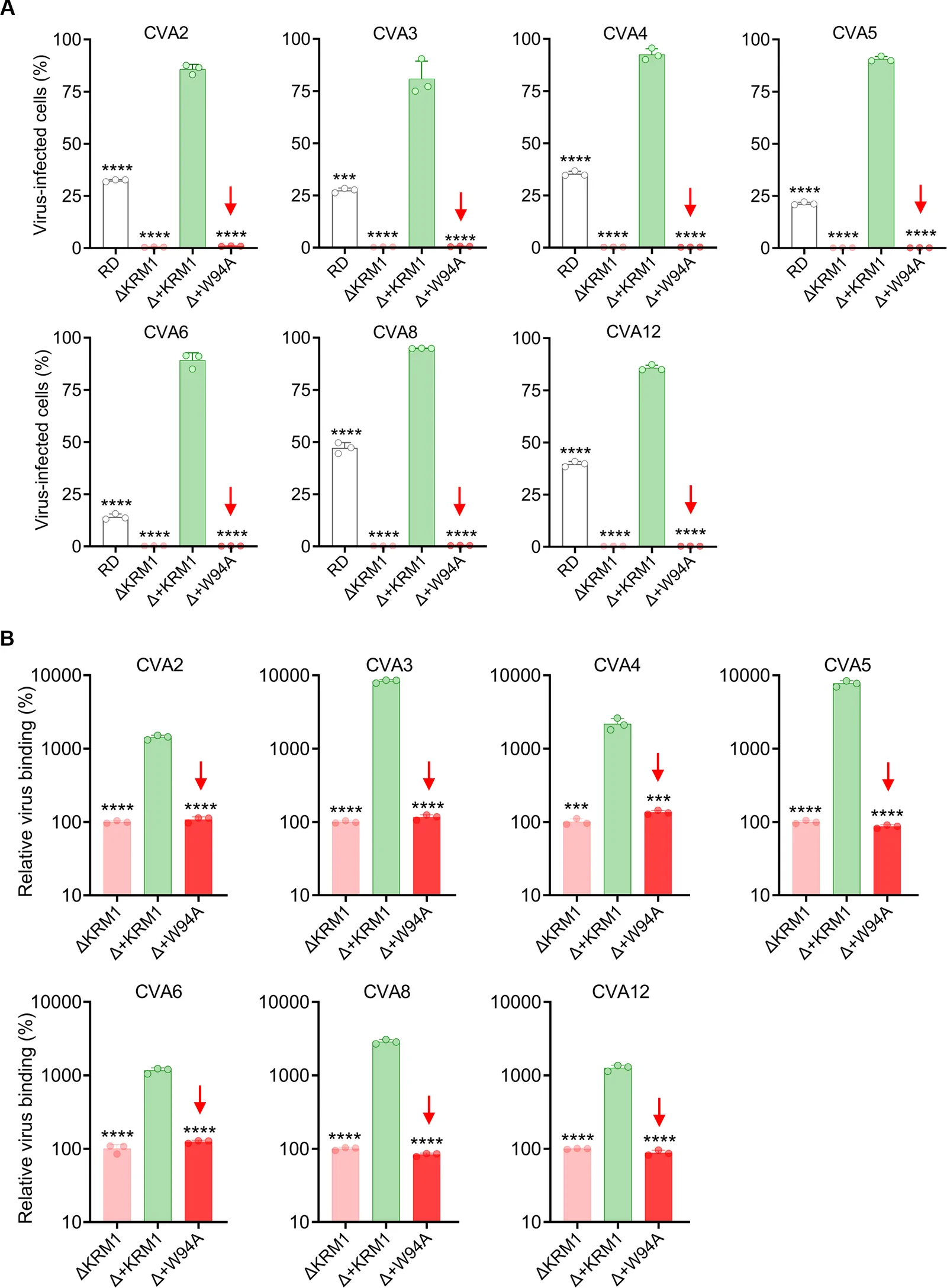
The KRM1 W94 residue is crucial for both the cellular binding and infection of all KRM1-dependent enteroviruses. (**A**) KRM1 W94 residue is essential for infection by KRM1-dependent enteroviruses. RD, ΔKRM1, Δ+KRM1, and Δ+W94 A cells were infected with the KRM1-dependent enteroviruses (MOI = 1) for 6 h. Cells were stained with the anti-dsRNA antibody, and virus-infected cells were analyzed by flow cytometry. Data are mean ± SD of three biological replicates. (**B**) Analysis of KRM1-dependent enterovirus binding to RD, ΔKRM1, Δ+KRM1, or Δ+W94 A cells by RT-qPCR. The CVA10 RNA bound to ΔKRM1 cells was defined as 100%. In this figure, each data point represents an individual well from a 24-well plate, corresponding to an individual replicate; data are mean ± SD of three biological replicates. Statistical significance was determined by two-tailed Student’s *t*-test between the KRM1-complemented ΔKRM1 group and the other groups. ***, *P* < 0.001; ****, *P* < 0.0001. Note that the downward arrows indicate that viral infection and binding were inhibited.

Having established that KRM1 W94 is essential for CVA10 infection, we next asked whether this residue is required for infection by other KRM1-dependent enteroviruses, including CVA2, CVA3, CVA4, CVA5, CVA6, CVA8, and CVA12. RD cells, ΔKRM1 cells, and ΔKRM1 cells stably reconstituted with wild-type KRM1 (Δ+KRM1) or the W94A mutant (Δ+W94A) were infected with each of these viruses (Fig. 6A). All viruses failed to infect ΔKRM1 cells, confirming their dependence on KRM1. Reconstitution with wild-type KRM1 fully restored infectivity for each virus tested. In striking contrast, expression of the W94A mutant did not rescue infection for any of these viruses (Fig. 6A).

To determine whether this defect arises from impaired virus-receptor binding, we performed cell-based attachment assays. The amount of virus bound to ΔKRM1 cells was set as 100%. Expression of wild-type KRM1 markedly enhanced binding of all tested viruses (10-fold to 100-fold over ΔKRM1 cells). In contrast, cells expressing the W94A mutant failed to increase binding, remaining at levels comparable to ΔKRM1 cells (Fig. 6B). These results demonstrate that the KRM1 W94 residue is essential for both cellular binding and infection of all KRM1-dependent enteroviruses tested.

### The KRM1 W94A mutation confers resistance to infection by all KRM1-dependent enteroviruses in mice

**Fig 7:**
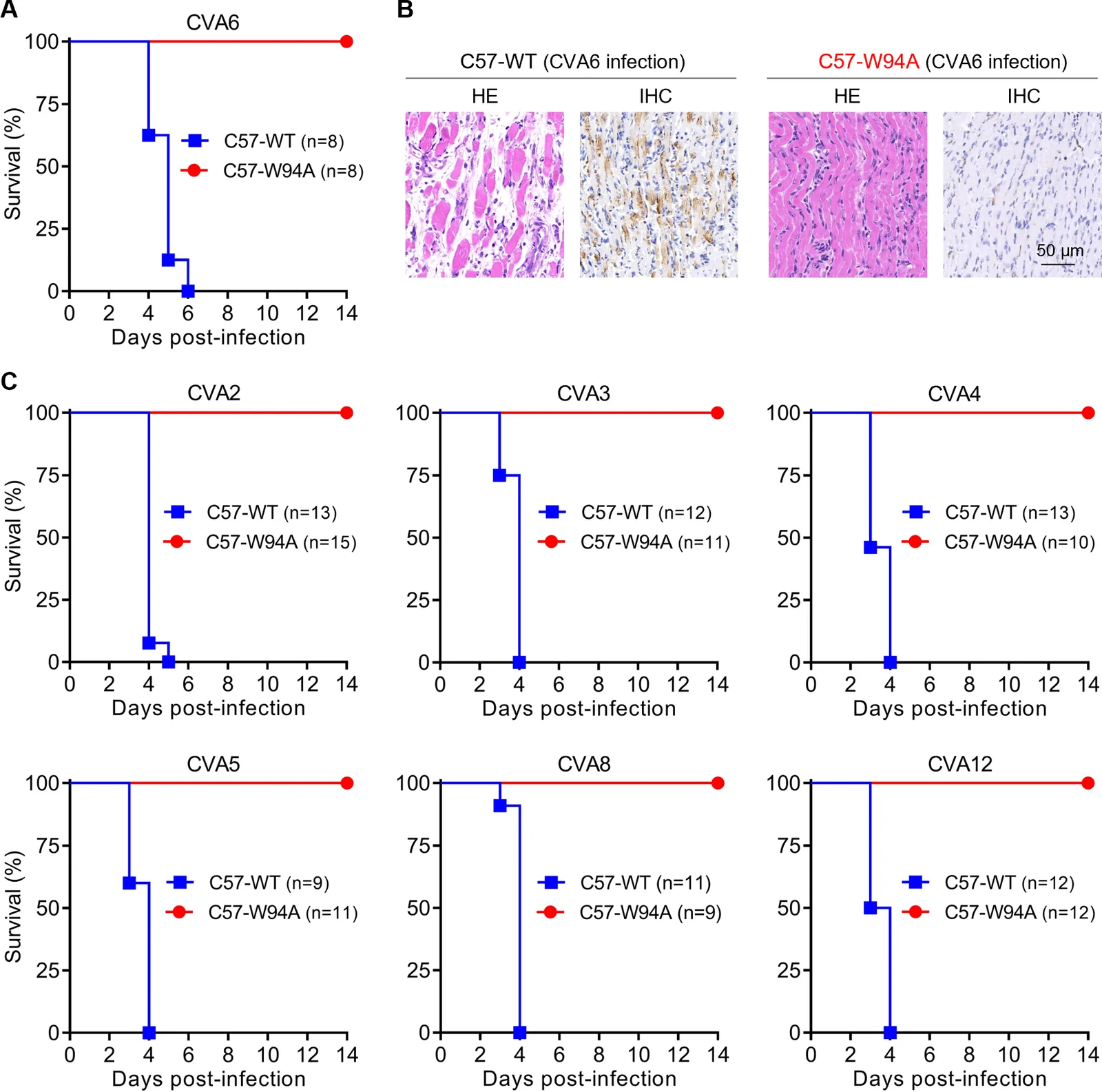
The KRM1 W94A mutation confers resistance to infection by all KRM1-dependent enteroviruses in mice. (**A**) Survival curves of C57-WT (blue line) and C57-W94A (red line) mice following intraperitoneal infection with CVA6 (100 TCID_50_). Group sizes (N) are indicated. (**B**) CVA6-infected mice were euthanized at 3 dpi, and limb muscle tissues were collected for H&E staining and immunohistochemical analysis using J2 antibody. Scale bar, 50 μm. (**C**) Survival curves of C57-WT (blue line) and C57-W94A (red line) mice following intraperitoneal infection with other KRM1-dependent enteroviruses. Survival data for mice represent the pooled statistical results from two independent experiments conducted under identical experimental conditions. Group sizes (*n*) are indicated.

We next evaluated whether the Krm1 W94A mutation protects against other KRM1-dependent enteroviruses *in vivo*. Given that CVA6 is a major cause of HFMD outbreaks worldwide, we first tested whether the Krm1 W94A mutation protects against CVA6 infection. Two-day-old C57-WT and C57-W94A mice were infected with CVA6. All C57-WT mice died within 4–6 dpi, whereas C57-W94A mice remained completely healthy and survived the observation period (Fig. 7A). At 3 dpi, limb muscle was collected for histopathological analysis. H&E staining of CVA6-infected C57-WT muscle showed severe myofiber necrosis, while C57-W94A muscle appeared normal (Fig. 7B). Immunohistochemical staining for dsRNA with J2 antibody revealed strong viral replication signals in C57-WT muscle, but no detectable signal in C57-W94A tissue (Fig. 7B).

We further challenged C57-WT and C57-W94A mice with CVA2, CVA3, CVA4, CVA5, CVA8, and CVA12. In all cases, C57-WT mice succumbed within 3–5 dpi, whereas C57-W94A mice exhibited complete resistance and survived without clinical signs (Fig. 7C). Collectively, these results demonstrate that the KRM1 W94 residue is indispensable for the pathogenesis of all KRM1-dependent enteroviruses *in vivo*.

## DISCUSSION

In this study, we identified KRM1 W94 as a critical receptor determinant for a major group of enteroviruses. W94 is essential for receptor binding and infection of these viruses, as its substitution with alanine abrogated both processes. Consistent with this, mice carrying the Krm1 W94A mutation were completely resistant to CVA10 and all other KRM1-dependent enteroviruses tested. Structural analysis revealed that W94 engages in a π-cation interaction with the universally conserved viral VP2 residue K140. Together, these findings uncover a conserved receptor-virus interaction that is essential for broad enterovirus infection.

### KRM1 W94 is essential for receptor binding and infection of a major group of enteroviruses

Through alanine scanning of 29 KRM1 residues at the CVA10-KRM1 interface, we identified W94 as the sole residue whose mutation (W94A) nearly abolished viral infection (Fig. 1E). At the biochemical level, W94A disrupted direct KRM1-virus binding in pull-down and ELISA assays (Fig. 3A and B). At the cellular level, it severely impaired viral attachment to the cell surface (Fig. 3E). These defects translated into complete resistance *in vivo*: CRISPR-engineered Krm1 W94A mice survived CVA10 infection without clinical signs or detectable viral replication, whereas wild-type mice succumbed (Fig. 4). Moreover, the W94A mutation conferred universal resistance to all other KRM1-dependent enteroviruses tested (CVA2–CVA6, CVA8, and CVA12), both in cell culture and in mice (Fig. 6 and Fig. 7). Collectively, these results establish KRM1 W94 as a critical and universal receptor determinant essential for both biochemical and cellular receptor binding and consequently for infection by this entire group of enteroviruses.

Our alanine scanning revealed that only W94 is essential among the 29 interface residues, whereas mutations in other residues had little effect individually (Fig. 1E). This suggests that the receptor relies on a single dominant hotspot, with other residues contributing weakly on their own and possibly in combination. Such a design favors viral evolution: most receptor-contacting sites can tolerate changes, allowing the virus to accumulate mutations for immune escape. At the same time, the virus still relies on the conserved anchor for receptor binding. If multiple receptor residues were each essential, viral diversification would be severely restricted. Thus, a single hotspot allows the virus to maintain stable receptor engagement while gaining the flexibility needed to evade host immunity.

### KRM1 W94 functions through pairing with the conserved VP2 residue K140

The W94A mutation abolished viral binding and infection (Fig. 1 and Fig. 3). Importantly, it did not alter KRM1’s overall structure or biochemical properties (Fig. 2), indicating a specific loss of function rather than global misfolding. Structural analysis of the CVA10-KRM1 complex revealed that W94 engages in a π-cation interaction with the viral VP2 residue K140 (Fig. 5F). We previously demonstrated that the VP2 K140 residue is completely conserved across all KRM1-dependent enteroviruses and plays an essential role in receptor binding and viral infection (11). The indole ring of W94 stacks against the positively charged side chain of K140, providing a critical energetic contribution to receptor recognition (Fig. 5F). Saturation mutagenesis further supported this model: only the aromatic substitutions W94F and W94Y partially retained infectivity, consistent with weaker π-cation interactions. In contrast, all other mutations, including W94A, completely abrogated function (Fig. 5). Thus, the W94-K140 pair represents the core hotspot at the virus-receptor interface, explaining why both residues are essential for broad enterovirus infection.

We have identified the W94-K140 pair as a conserved anchoring hotspot within KRM1-dependent enteroviruses. This raises a question: do similar mechanisms exist in other enterovirus receptor systems? For instance, coxsackievirus B1–B6 share CAR as a common receptor (16, 17), and multiple echoviruses use FcRn (18); however, it is unclear whether conserved viral residues act as universal anchors for these receptors or whether corresponding receptor hotspots similarly govern broad enterovirus serotype recognition. Answering these questions will require structural analyses of virus-receptor complexes, combined with sequence conservation and mutagenesis studies.

### Methodological implications: from structural predictions to cellular validation

This study provides a systematic strategy for identifying essential receptor residues, with implications for analogous virus-receptor studies. Structural and biochemical analyses initially predicted multiple KRM1 residues (D88, D90, W94, and W106) as CVA10 contacts, and alanine substitutions at all four abolished direct binding *in vitro* (Fig. 3A and B). However, only W94A impaired viral attachment and infection in the cellular context (Fig. 1E and 3E). This discrepancy suggests that biochemical binding assays may overestimate the contribution of certain residues, as they cannot fully reproduce the native membrane environment, receptor clustering, or multivalent virus binding. Therefore, cell-based validation is essential for identifying true functional determinants. Our integrated approach combines structure, biochemistry, and cell-based attachment assays and offers a valuable framework for future studies of other virus-receptor interactions.

### Conclusion

In summary, this work identifies KRM1 W94 as the essential receptor determinant for a major group of enteroviruses. W94 is required for receptor binding and infection at the biochemical, cellular, and *in vivo* levels, functioning through a π-cation interaction with the universally conserved viral VP2 residue K140. Strikingly, Krm1 W94A mutant mice were completely resistant to infection, demonstrating that a single host amino acid change confers broad protection against multiple pathogens. These findings uncover a conserved virus-receptor hot spot, establish a mechanistic framework for broad enterovirus recognition, and provide a rationale for developing pan-enterovirus preventive and therapeutic strategies.

## MATERIALS AND METHODS

### Cells and viruses

Human malignant embryonic rhabdomyosarcoma (RD), human hepatocellular carcinoma Huh-7, and HEK 293T cells were maintained in Dulbecco’s modified Eagle’s medium (DMEM, Corning, NY, USA) supplemented with 10% heat-inactivated fetal bovine serum (FBS), 100 U/mL penicillin, and 100 μg/mL streptomycin at 37°C in a humidified atmosphere containing 5% CO₂.

The following viruses were used in this study and have been described previously (11): CVA2 strain HN202009 (HN; GenBank ID: MT992622), CVA3 strain XZ99086-1999 (XZ; National Microbiology Data Center [NMDC] ID: NMDCN0003ADI), CVA4 strain High Point (HP; GenBank ID: AY421762), CVA5 strain 2023ZZ285 (2023ZZ285; NMDC: NMDCN0007O5O), CVA6 strain 54203/HeB/CHN/2012 (HeB; GenBank ID: MK106189), CVA8 strain 14-943/GS/CHN/2014 (GS14; GenBank ID: MT648781), CVA10 prototype strain Kowalik (GenBank ID: AY421767), and CVA12 strain Texas-12 (Texas; GenBank ID: AY421768). Viral titers were determined by the 50% tissue culture infectious dose (TCID_50_) assay on RD cells.

### Proteins and antibodies

The pcDNA3.4-IL10-hKRM1-Fc-His plasmid, encoding the ectodomain (residues A23–G373) of KRM1, has been described previously (15). KRM1 mutants (D88A, D90A, W94A, Y105A, W106A, and Y108A) were subsequently introduced using PCR-based site-directed mutagenesis. The resulting plasmids were transiently transfected into 293T cells using a commercial transfection kit (Youyi, YYTrans.K01) according to the manufacturer’s protocol. Culture supernatants were harvested 7 days post-transfection, and recombinant His-tagged hKRM1-Fc proteins were purified using Ni Sepharose FF (Vazyme, HP202-01). The purified KRM1-Fc-His and its mutant proteins were quantified by the Bradford assay, aliquoted, and stored at −80°C.

Rabbit polyclonal antibodies against CVA10 VP0 were raised according to established protocols (19), using *E. coli*-expressed VP0 proteins of the Kowalik strain as immunogens.

### Construction of KRM1 single-amino-acid mutant plasmids

DNA fragments harboring desired point mutations within the KRM1 coding sequence were synthesized and cloned into a pUC vector (Genewiz, Suzhou). After PCR amplification, these fragments were inserted into the pCMV3-KRM1-Flag backbone via homologous recombination, replacing the corresponding wild-type region. All constructs were confirmed by Sanger sequencing. Two additional mutants, KRM1 D88K and D90K, were generated by PCR-based site-directed mutagenesis using the wild-type pCMV3-KRM1-Flag plasmid as the template. A saturation mutagenesis library targeting the W94 residue of KRM1 was also constructed by GenScript (Nanjing, China).

### Viral infection assay

For single-round infection assays, transfected cells were washed once with PBS and infected with CVA10 at an MOI of 1 at 37°C for 1 h. Then, the inoculum was removed, the cells were washed three times with PBS, and fresh culture medium was added. At 6 h post-infection, cells were harvested for flow cytometric analysis of viral infection. Briefly, cells were fixed, permeabilized, and incubated for 30 min at room temperature with 50 μL of wash buffer containing the anti-dsRNA J2 antibody (CST, 76651). After washing to remove unbound antibodies, cells were further incubated for 20 min at room temperature with 100 μL of the wash buffer containing APC-conjugated anti-DYKDDDDK antibody (BioLegend, 637308) and donkey anti-mouse IgG (H + L) secondary antibody (Invitrogen, R37114). Following a final wash, the samples were analyzed by flow cytometry. The infection rate was defined as the proportion of dsRNA-positive cells within the Flag-positive cell population (Fig. S3). Non-transfected ΔKRM1 cells were used to establish the Flag-negative gate, and ΔKRM1 cells transfected with pCMV3-hSCARB2-Flag served as the dsRNA-negative control.

For multi-round infection assays, the cells were infected with CVA10 (MOI = 0.01) for 1 h, washed, and cultured for 24 h. The cells and supernatants were then subjected to two freeze-thaw cycles, and viral titers were determined by TCID_50_ assay.

### Generation of Krm1 W94A mutant mice

The *Krm1* W94A mouse model was established on a C57BL/6J background using a CRISPR/Cas9-mediated homologous recombination system. Two single-guide RNAs (sgRNA-1: CGTGAGCCCTTGGTGCTACG; sgRNA-2: GCTCGGCCACGTAGCACCAA) were designed to target the genomic sequences adjacent to the tryptophan 94 codon (TGG) in the mouse Krm1 gene (Transcript ID: NM_032396.3). A single-stranded oligodeoxynucleotide (ssODN) containing the desired TGG-to-GCG point mutation was synthesized as the homologous recombination donor template. A synonymous mutation was simultaneously introduced to disrupt the original sgRNA recognition site (thereby preventing re-cleavage) and to create a novel *AatII* restriction site for subsequent genotyping.

The mixture of Cas9 mRNA, the selected sgRNA, and the ssODN donor template was microinjected into the pronuclei of fertilized C57BL/6 mouse zygotes. To identify potential founder (F0) mice, the targeted genomic region was amplified by PCR using the primers Krm1-ident-IN-F (5′-TTTCCTCCCTGAAGAGCGATAA-3′) and Krm1-ident-IN-R (5′-GCCAGTCTCAGAAAACAAGGTG-3′). The PCR products were initially screened using a T7 endonuclease I (T7E1) assay to detect the presence of indel mutations. Positive samples were then subjected to restriction fragment length polymorphism (RFLP) analysis using *AatII* to detect the successful incorporation of the donor template. The precise mutation was subsequently confirmed by TA-cloning of the PCR products followed by Sanger sequencing. Founder (F0) mice identified by Sanger sequencing were bred through successive generations to establish a homozygous Krm1 W94A mouse line.

### Generation of KRM1-knockout RD cell lines

To generate KRM1-knockout RD cells, two sgRNAs targeting exon 2 of *KRM1* (sgRNA-1: AACTGGACAGCACTACAAGG; sgRNA-2: CACTCTGAAATACCCCAACG) were commercially synthesized. Ribonucleoproteins (RNPs) were assembled by incubating the synthesized sgRNAs with Cas9 protein (GenScript, Z03702) at 37°C for 20 min and were then delivered into RD cells using the Lonza 4D-Nucleofector system according to the manufacturer’s protocol (Lonza, V4XC-2032). Following CRISPR-mediated editing, a pooled population of KRM1 knockout (KO) RD cells was subjected to limiting dilution to isolate single-cell clones. Because KRM1 is the established receptor for CVA10, each clone was subsequently infected with CVA10 to assess viral susceptibility. Clones exhibiting the strongest resistance to CVA10 infection were selected for a second round of single-cell cloning, thereby ensuring the establishment of a genetically homogeneous KRM1-deficient cell line.

To verify CRISPR-induced mutations at the genomic level, genomic DNA from the selected KRM1 KO clones was extracted and used as a template for PCR amplification of KRM1 exon 2. The resulting amplicons were ligated into a TA cloning vector and analyzed by Sanger sequencing. To further validate gene disruption at the transcriptional level, KRM1 mRNA expression was quantified by reverse transcription quantitative PCR (RT-qPCR). Briefly, total RNA was extracted from both wild-type and KRM1 KO RD cells using the Super FastPure Cell RNA Isolation Kit (Vazyme, RC102) following the manufacturer’s instructions. cDNA was synthesized using the HiScript II 1st Strand cDNA Synthesis Kit (Vazyme, R211). Quantitative PCR reactions were performed using the ChamQ Universal SYBR qPCR Master Mix (Vazyme, Q312) with primers specific for KRM1 and β-actin. The following primer sequences were used: KRM1-F: 5′-GACAGCACTACAAGGCGGG-3′; KRM1-R: 5′-TCCCCGTTGGGGTATTTCAG-3′; β-actin-F: 5′-GGACTTCGAGCAAGAGATGG-3′; β-actin-R: 5′-AGCACTGTGTTGGCGTACAG-3′. KRM1 mRNA levels were normalized to β-actin mRNA levels.

### Generation of cells stably expressing KRM1-WT and KRM1-W94A

The full-length human *KRM1*-coding sequence was cloned into the pHAGE-CMV-Flag-puro lentiviral vector via homologous recombination using the ClonExpress II One Step Cloning Kit (Vazyme, C112), generating the construct pHAGE-CMV-hKRM1-Flag-puro. The W94A mutation was subsequently introduced into the same vector backbone to generate pHAGE-CMV-hKRM1-W94A-Flag-puro.

For lentivirus production, each construct was co-transfected with pVSV-G and psPAX2 plasmids into 293T cells at a ratio of 1:1:3 using standard transfection procedures. After 48 h, the culture supernatants were collected, clarified by centrifugation, and filtered through a 0.22-μm membrane to obtain lentiviral stocks.

To establish stable KRM1-expressing cell lines, ΔKRM1 cells were seeded in six-well plates and transduced with 1 mL of lentiviral supernatant mixed with 1 mL of DMEM containing 2% FBS. After 12 h of incubation, the medium was replaced with fresh medium. Two days post-transduction, the cells were subjected to puromycin selection (2 μg/mL) for 2 weeks. The surviving cell populations, stably expressing either KRM1-WT or KRM1-W94A, were designated as Δ+KRM1 and Δ+W94A, respectively. Using the same approach, huh-7 cells were transduced with the lentiviruses, yielding Huh-7+KRM1 and Huh-7+W94 A cell lines.

To confirm KRM1-Flag protein expression in these cells, intracellular Flag staining was performed and analyzed by flow cytometry. Briefly, 2 × 10⁵ cells from each line were fixed, permeabilized, and washed using BD Perm/Wash Buffer (BD, 554723), followed by staining with APC-conjugated anti-DYKDDDDK (Flag) antibody for 30 min at room temperature. After washing with Perm/Wash buffer, fluorescence signals were analyzed by flow cytometry to verify intracellular expression of Flag-tagged KRM1.

### *In vivo* infection assay

To evaluate the role of the W94 residue in KRM1-dependent enterovirus infection *in vivo*, groups of 2-day-old C57-WT and C57-W94A mice were inoculated intraperitoneally (i.p.) with PBS or with different viruses at the following doses: CVA2, CVA5, CVA10, and CVA12 at 100,000 TCID_50_ per mouse; CVA3 at 50,000 TCID_50_ per mouse; CVA4 at 1,000 TCID_50_ per mouse; CVA6 at 100 TCID_50_ per mouse; and CVA8 at 3,000 TCID_50_ per mouse. Mice were monitored daily for clinical symptoms and mortality over a 14-day period.

For pathological analysis, groups of infected mice were euthanized at 3 dpi. Limb muscles were collected, fixed in 4% paraformaldehyde, and stained with hematoxylin and eosin (H&E) (Servicebio, China). For immunohistochemical analysis, limb muscles collected at the indicated time points were fixed, embedded, sectioned, and subsequently stained with the J2 antibody (CST, 76651) to assess viral replication (Servicebio, China).

### Coomassie staining

KRM1-Fc-His and its mutant protein samples were denatured by mixing with Omni-Easy Instant Protein Loading Buffer (Epizyme, LT101) and subsequently boiled at 104°C for 5 min. The samples were then centrifuged at 12,000 rpm for 3 min, and the supernatants were run on 4%–20% YoungPAGE gels (GenScript, M01004) at 120 V for 80 min. After electrophoresis, the gels were stained with Coomassie Rapid Staining Solution (Epizyme, PS111) for 10 min and then washed three times with H_2_O, with each wash lasting 10 min. The stained gels were imaged using a ChemiDoc Imaging System (Bio-Rad).

### Pull-down assay

Purified SCARB2-Fc-His, KRM1-Fc-His, or the indicated KRM1 mutant proteins (2 μg each) were incubated with 5 × 10⁷ TCID_50_ of CVA10 at 4°C for 2 h to allow sufficient binding between viral particles and the receptor proteins. Subsequently, 20 μL of pre-equilibrated Ni Smart Magarose Beads (Smart-Lifesciences, SM025005) was added to each mixture, followed by rotation at 4°C overnight. The beads were collected using a magnetic stand, and the supernatant was discarded. The beads were washed three times with wash buffer containing 5 mM imidazole to remove nonspecific binding. Bound proteins were eluted with 100 μL of elution buffer by gentle pipetting, and the eluates were collected after bead separation. Samples were then separated by SDS-PAGE and analyzed by western blotting. SCARB2-Fc-His, KRM1-Fc-His, and the mutant proteins were detected using an anti-His tag antibody (Proteintech, 66005-1-Ig), while CVA10 was detected using the anti-CVA10 sera described above.

### Receptor protein neutralization assay

RD cells were seeded in 24-well plates and cultured overnight. KRM1-Fc-His or the indicated KRM1 mutant proteins (5 μg/mL final concentration) were mixed with 2 × 10⁵ TCID_50_ of CVA10 and incubated at 37°C for 1 h. The mixtures were then added to RD cells and allowed to infect at 37°C for 1 h. The inoculum was removed, and cells were washed three times with PBS before fresh culture medium was added. At 6 h post-infection, cells were harvested, and the percentage of CVA10-positive cells was determined by flow cytometry as described above.

### Virus attachment assay

ΔKRM1, Δ+KRM1, Δ+W94A, or ΔKRM1 cells transfected with the indicated cDNAs were pre-chilled at 4°C. Pre-cooled DMEM containing 1 × 10⁶ TCID_50_ of virus was then carefully added to the cells, followed by incubation on ice for 1 h to allow viral binding. Cells were gently washed three times with ice-cold PBS to remove unbound virus. Cells were then lysed, and total intracellular RNA was extracted as described above. Purified RNA was reverse-transcribed into cDNA, and quantitative PCR (qPCR) was performed to detect viral RNA and β*-actin* mRNA. The primer sequences for KRM1-dependent enterovirus detection are listed in Table S1. All data were analyzed using the 2⁻^ΔΔCt^ method, and viral RNA levels were normalized to β*-actin* mRNA levels.

### CVA10 binding ELISA

ELISA plates were coated with 100 ng/well of purified CVA10 at 4°C overnight and then blocked with 5% milk in PBST for 2 h. The plates were then incubated with 50 μL/well of serially diluted KRM1-Fc-His or KRM1 mutant proteins at room temperature for 2 h. After five washes with PBST to remove unbound proteins, the plates were incubated with HRP-conjugated goat anti-human IgG (Proteintech, SA00001-17) for 1 h at room temperature. Following another wash, TMB substrate solution (NCM, M30100) was added to each well for color development, and the reaction was terminated by adding 2 M H₂SO₄. The absorbance was measured at 450 nm using a microplate reader.

### Circular dichroism (CD) spectroscopy

The far-UV circular dichroism (CD) profiles of the protein samples were recorded on a Jasco J-1500 spectropolarimeter. Measurements were conducted at ambient temperature, scanning from 190 to 260 nm at a speed of 50 nm/min. A quartz cell with an optical path length of 0.1 cm was used for all experiments, with the protein concentration maintained at 100 μg/mL.

### High-performance liquid chromatography (HPLC)

KRM1-Fc-His and KRM1 mutant proteins were analyzed based on their intrinsic hydrophobicity using an Agilent 1260 Infinity II HPLC system equipped with a short C3 reverse-phase column. Separation was achieved under a gradient of increasing organic solvent. The distinct elution profiles, monitored at 280 nm, provided characteristic fingerprints for each protein, with retention times directly correlating to their relative hydrophobicity.

### Structural analysis of the interaction between CVA10 and KRM1

The cryo-EM structure of the CVA10-KRM1 complex (12) was used for structural analysis. The structural representations were generated using UCSF Chimera software (v1.15) (20).

## Data Availability

All relevant data are provided as figures within the paper. Other data are available from the corresponding authors upon reasonable request.

## SUPPLEMENTAL MATERIAL

Supplemental material mbio.01123-26-s0001.pdf Fig. S1-S3; Table S1.

ASM does not own the copyrights to Supplemental Material that may be linked to, or accessed through, an article. The authors have granted ASM a non-exclusive, world-wide license to publish the Supplemental Material files. Please contact the corresponding author directly for reuse.
