# Supplementary material for: KREMEN1 residue W94 is essential for receptor binding and infection by a major group of enteroviruses
Source: mBio. 2026 Jul 31;17(9):e01123-26. doi: 10.1128/mbio.01123-26 (PMC13556202; doi:10.1128/mbio.01123-26)
Supplement: Supplementary file 1 [file mbio.01123-26-s0001.pdf]

Supplementary information for

KREMEN1 residue W94 is essential for receptor binding and infection by a major group of enteroviruses

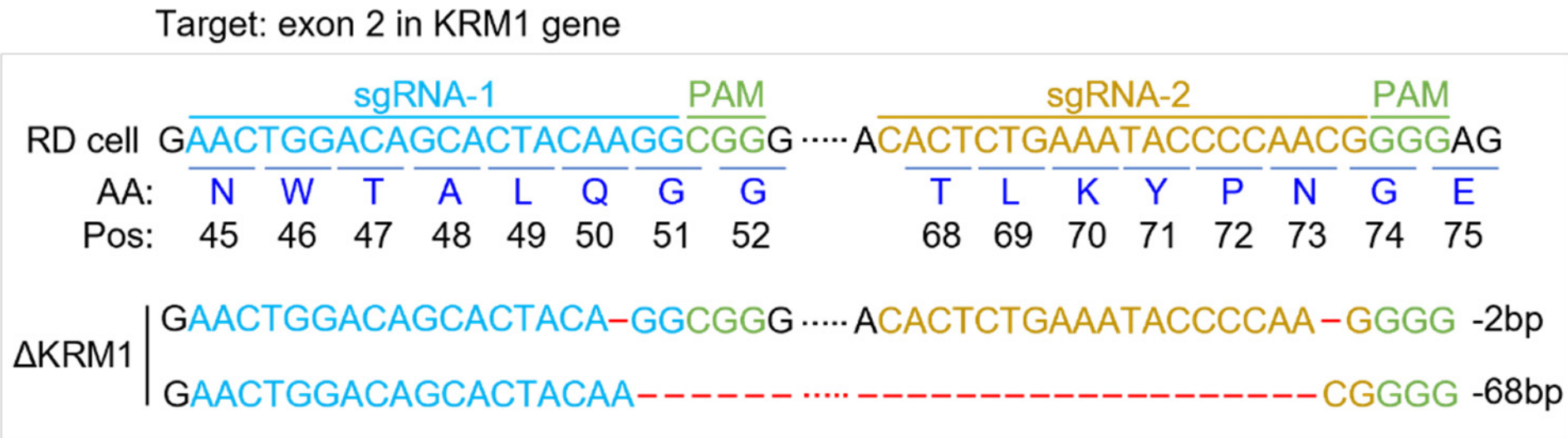

Figure S1. Sanger sequencing confirmed deletions in the sgRNA-targeted region in ΔKRM1.

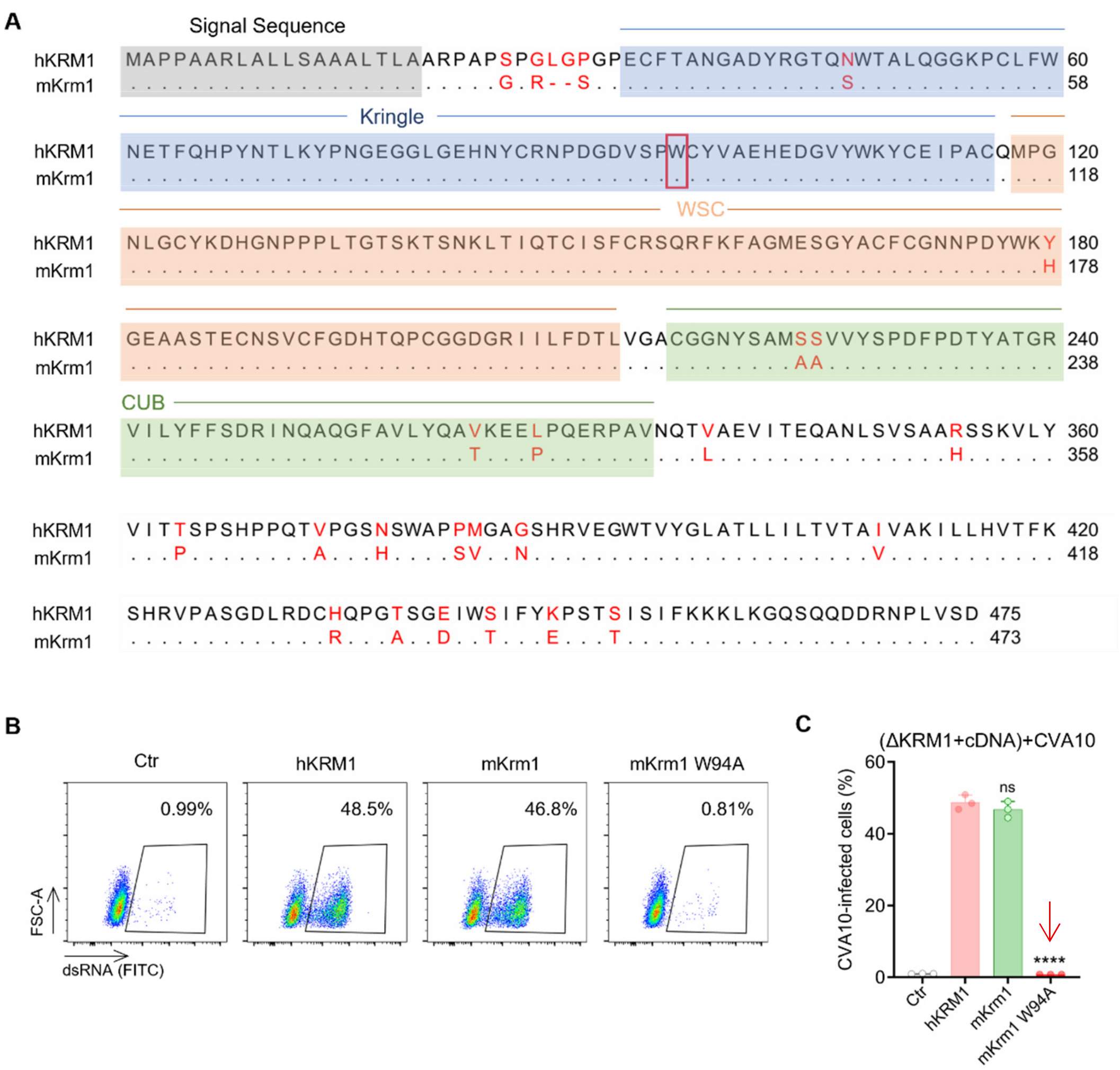

Figure S2. Sequence alignment and functional analysis of hKRM1 and mKrm1.

(A) Sequence alignment of hKRM1 and mKrm1 proteins. The signal peptide, Kringle, WSC, and CUB domains of the KRM1 protein are indicated in light gray, light blue, light yellow, and light green, respectively. Red shading indicates non-conserved residues between the two species, and the red box highlights the KRM1 W94 residue.

(B-C) ΔKRM1 cells were transfected with cDNA of SCARB2 (Ctr), hKRM1, mKrm1 and mKrm1 W94A, then infected with CVA10 at an MOI of 1 for 6 hours. The cells were stained with J2 antibody and the percentage of CVA10-infected cells was analyzed by flow cytometry. Downward arrow indicates the mKrm1 W94A substitution that impaired CVA10 infection. **(B)** Representative results; **(C)** Statistical results from three independent replicates. Data are mean ± SD from three biological replicates. Statistical significance was determined by two-tailed unpaired t-test. ****, $P < 0.0001$; ns, not significant ($P \geq 0.05$).

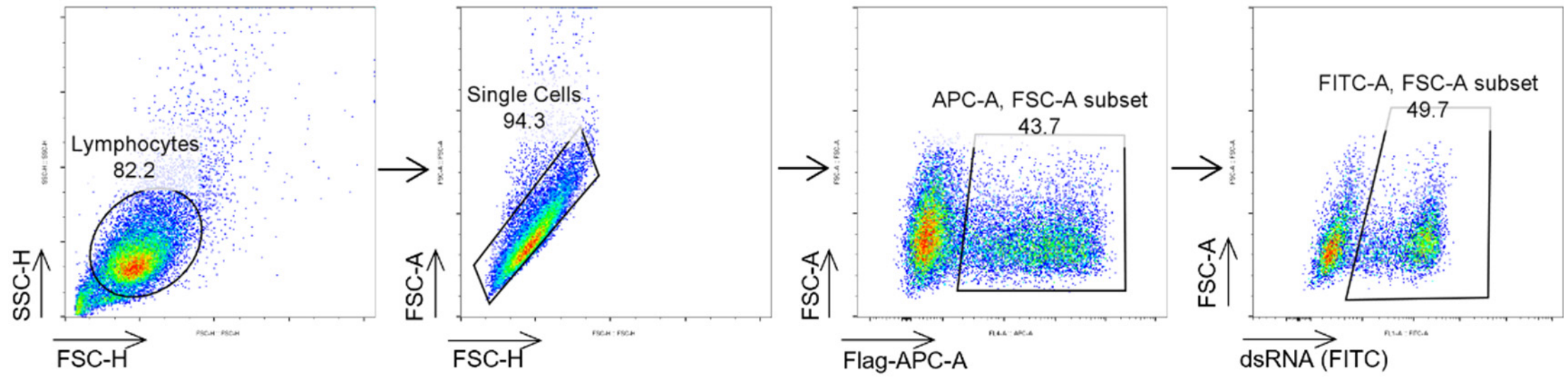

Figure S3. Gating strategy for quantification of viral infection rates.

Table.S1. qPCR primers used for detection of viral RNA.

Name	Sequence (5'-3')
CVA2-F	TGTATGTCCCACCTGGAGCAC
CVA2-R	AATGCGTTGTTGGGGCATTG
CVA3-F	TGTGAGTGAGCACAGTATAGGCAC
CVA3-R	ACTCTGCATTGAAGCGCATGT
CVA4-F	GGGCAATGTCCCAATAATATGCTGG
CVA4-R	TCTTGCGCCTATCTTTGGCC
CVA5-F	GAGGTCCCGGCCACTACTAATC
CVA5-R	AGTCCTGTTAGATGAAGCGTCCAC
CVA6-F	ACAGTGTCCTAACAACATGATGGGAC
CVA6-R	GGTAGTTATGCTTGCACGGTCG
CVA8-F	ACGGCCAGTGCCCTAACAATATG
CVA8-R	GTGGAGTCTATCTTGGCCCCATC
CVA10-F	GAAATGGAGTGTTGGAGGCGA
CVA10-R	TTTCTGTTGTAGTGACGAATG
CVA12-F	CCAGCTAGTGCATACCAATGGTTC
CVA12-R	GCTTCTAATTGGGCGAGGCAC
